# Wording Effects and the Factor Structure of the Hospital Anxiety & Depression Scale in HIV/AIDS Patients on Antiretroviral Treatment in South Africa

**DOI:** 10.1371/journal.pone.0034881

**Published:** 2012-04-20

**Authors:** Edwin Wouters, Frederik le Roux Booysen, Koen Ponnet, Francis Baron Van Loon

**Affiliations:** 1 Department of Sociology and Research Centre for Longitudinal and Life Course Studies, University of Antwerp, Antwerp, Belgium; 2 Centre for Health Systems Research and Development, University of the Free State, Bloemfontein, Republic of South Africa; 3 Department of Economics, University of the Free State, Bloemfontein, Republic of South Africa; 4 Media & ICT in Organisations and Societies (MIOS), University of Antwerp, Antwerp, Belgium; University of Washington, United States of America

## Abstract

**Background:**

Given the immense burden of HIV/AIDS on health systems in sub-Saharan Africa and the intricate link between HIV/AIDS and mental health problems, health care providers need a valid and reliable instrument to assess mental health rapidly. The Hospital Anxiety and Depression Scale (HADS) may constitute such an instrument. The aims of this study were to: (1) examine the factor structure of the HADS in a population of South African HIV/AIDS patients on antiretroviral treatment (ART); and (2) identify and control the disturbing influence of systematic wording effects in vulnerable respondent groups.

**Methodology/Principal Findings:**

The translated scale was administered to 716 HIV/AIDS patients enrolled in the public sector ART program in South Africa. A combined confirmatory factor analysis and correlated-traits-correlated-methods framework was used to determine the preferred factor structure of the HADS, while controlling for the disturbing influence of systematic wording effects. When assessing the structure without a negative wording factor, all three factor structures displayed an acceptable fit to the data. The three-factor solution best fitted the data. Addition of a method factor significantly improved the fit of all three factor solutions. Using χ^2^ difference testing, Razavi's one-factor solution displayed a superior fit compared to the other two factor solutions.

**Conclusions:**

The study outcomes support the use of the HADS as a valid and reliable means to screen for mental health problems in HIV/AIDS patients enrolled in a public-sector ART program in a resource-limited context. The results demonstrate the importance of evaluating and correcting for wording effects when examining the factor structure of the screening instrument in vulnerable patient groups. In light of the inter-relationships between HIV/AIDS and mental health problems and the scarcity of adequate screening tools, additional studies on this topic are required.

## Introduction

According to the World Health Organization, HIV/AIDS (3) and depression (8) are among the 10 leading causes of the disease burden in the developing world [1]. Low- and middle-income countries bear the bulk of the HIV/AIDS burden. Sub-Saharan Africa, the poorest region in the world, has been hit hardest by HIV/AIDS, accounting for 68% of all people living with HIV and for 72% of AIDS deaths in 2009 [2]. In terms of disease-adjusted life years, low- and middle-income countries also bear a considerably greater share of the burden of depression than do high-income countries [1].

It has been established that HIV/AIDS and depressive disorders are intricately interlinked. First, depression and anxiety hamper HIV prevention: research has demonstrated that mental health problems are associated with high-risk sexual behavior (e.g. inconsistent condom use) and may contribute to increased HIV transmission [3]–[5]. Second, HIV-positive diagnosis is a stressor that has been found to increase significantly the chance of depression and anxiety [6], [7]. In turn, several studies have suggested that depressive and anxiety disorders may worsen HIV-related health outcomes, and antiretroviral treatment (ART) outcomes in particular. Depression and anxiety have been associated with poor ART adherence, declines in CD4 counts, rapid progression to AIDS, and increased mortality [8]–[11].

The vast majority of scientific research on the link between depressive and anxiety disorders and HIV/AIDS has been performed in western industrialized settings, and only a few studies have examined these mental illnesses among treated HIV-infected individuals in sub-Saharan Africa [8], [12]. However, given the high HIV prevalence and the limited human and monetary resources in the region, it is crucial to identify and treat depressive and anxiety disorders effectively to use optimally the limited resources available for antiretroviral care. Given the immense burden that HIV/AIDS and the associated antiretroviral care are putting on the health system, nurses need a valid and reliable instrument to assess rapidly the mental health of each patient. The Hospital Anxiety and Depression Scale (HADS) has been extensively used as both a clinical and a research tool and could therefore be the much needed tool to assess mental health problems in high-HIV-prevalence, resource-limited settings. This self-report screening instrument is quick to use and acceptable to patients who may feel unwell, because it only consists of 14 items each answered on a four-point verbal scale [13], [14].

Fundamental to the screening efficacy of the HADS is that it validly and reliably measures well-defined dimensions of mental health. The factor structure of the HADS has been extensively investigated in various populations. The HADS was originally developed by Zigmond and Snaith in 1983 to identify symptoms of depression (seven items) and anxiety (seven items); a factor structure supported by the majority of studies using an exploratory approach [15]–[18]. However, some studies have proposed alternative factor structures, including a one-factor structure [13], [19] to measure emotional distress, or a three-factor solution to measure depression, anxiety and negative affect [20]–[24]. The vast majority of these studies, however, have been performed in western settings. Very few published studies have adopted the HADS into African languages [12]. In addition, only a limited number of studies has applied the HADS to assess depressive and anxiety disorders among HIV-infected individuals. An extensive literature review has revealed only one study that has assessed the factor structure of the HADS in a population of HIV-infected individuals in sub-Saharan Africa. Reda has reported a single underlying dimension as indicated by Razavi's model [12], [19]. The dearth of scientific literature on the factor structure of the HADS in high-HIV-prevalence, resource-limited settings renders this topic a research priority.

One must however note that, in the discussion of the factor structure of the HADS, the potential impact of methods effects associated with negatively worded items is often overlooked [18]. A series of studies that has predominantly examined the Rosenberg Self-Esteem scale, has demonstrated the existence of method effects associated with negatively and/or positively worded items, which can be interpreted as a response style [18], [25]–[28]. This increasing body of knowledge suggests that the inclusion of both positive and negative item phrasing creates a source of variance that can reduce the reliability and confound the factor structure of a scale [28], [29]. Tomás and Oliver [30] and more recently, DiStefano and Motl [27] have urged future research to study whether this wording effect is also present in other socio-psychological scales, and whether this effect is present across different ages and educational levels.

The HADS contains both positively and negatively formulated items to reduce acquiescent bias. However, as mentioned above, such a balanced scale creates the need to examine the presence of item wording effects to be able to ascertain the preferred factor structure of the HADS in our population. We thus need to examine whether a better model fit can be achieved by controlling each of the three proposed factor structures – the one-, two-, and three-factor structure – for item wording effects. This additional investigation is vital because recent studies have indicated that such a method effect factor is not a mere methodological artifact, but is also representative of a response style. It has been suggested that different sociocultural populations may respond differently to negatively (or positively) worded items with poorer, younger and less-educated people being more susceptible to wording effects [31]–[35], underlining the need to incorporate these method effects in exploring the factor structure of the HADS in vulnerable respondent groups such as HIV-infected patients in developing countries [32]. In this way, the current study responds to the research needs mentioned in the literature [27], [30].

The current study thus had two inter-related objectives. First, it aimed to address the above-cited research gap by examining the factor structure of the HADS in a sample of 716 HIV-infected individuals receiving public-sector ART in the Free State Province of South Africa. Second, the study aimed to detect and control for methods effects associated with negatively and/or positively worded items using Marsh & Grayson's Correlated Traits, Correlated Methods (CTCM) framework [36], which uses a structural equation modeling approach to model this wording effect as a latent trait [33], [37]. In this way, we aimed to determine the preferred factor structure of the HADS in this highly relevant population, while controlling for the disturbing influence of systematic wording effects in particularly vulnerable patient groups.

## Methods

This study is part of a prospective cohort study of patients enrolled in the public-sector ART program in the Free State Province of South Africa entitled, “Effective Aids Treatment and Support in the Free State (FEATS)”. The study is conducted by the Centre for Health Systems research and Development (CHSR&D) of the University of the Free State (UFS). The study was approved by the Ethics Committee of the Faculty of Health Science of the UFS [ETOVS 145/07 DOH-27-0907-2025] and authorized by the Provincial Department of Health.

### Study population

The first step was to recruit patients on ART into the study. As antiretroviral nursing staff at facilities interacts directly with ART patients, antiretroviral nurses were asked to assist in the recruitment. To yield statistically significant outcomes, 716 participants were recruited – by the nurses – into the study from 12 public ART clinics across five districts in the Free State Province of South Africa in 2007/08. Inclusion criteria were: age ≥18 years; having commenced ART in the past 5 weeks; and residing in the town or village where the particular health facility was located. The nurses provided potential participants with the relevant information and obtained written informed consent. Following the recruitment of ART patients into the study by nursing staff at the twelve selected ART facilities, trained interviewers conducted face-to-face interviews, using a standard questionnaire, only after obtaining written consent from all the participants for the second time.

### Questionnaire

The HADS was originally developed to detect depression and anxiety states among patients in non-psychiatric hospital clinics [14]–[16], [38]. The instrument consists of 14 items, both positively and negatively worded, and each rated on a four-point Likert scale indicating absence, possible presence (two categories) or probable presence of mental health problems. The questionnaire was translated from English to Sesotho independently by two researchers working at the CHSR&D, whose mother tongue was Sesotho, and these translations were checked by the Sesotho-speaking interviewers during training to check the acceptability and clarity of the items and the scale as a whole. The final translated instrument reflected the consensus on the wording, clarity and cultural equivalence of the individual items.

### Data analysis

To explore the data, we examined the HADS item distributions using SPSS version 16. Subsequently, we aimed to examine the factor structure of the HADS in our sample of 716 HIV-infected individuals receiving public-sector ART in the Free State Province of South Africa.

The current study uses confirmatory factor analysis (CFA) to compare the fit of the three alternative factor structures supported by the literature. The original two-factor model, developed by Zigmond and Snaith in 1983, discerned an anxiety and a depression subscale (seven items each) [15]. Alternatively, a number of studies have proposed a one-factor solution, in which all 14 items measure one dimension, namely emotional distress [12], [13], [19]. This structure has recently been supported by Reda in a population of Ethiopian HIV-infected patients [12]. Recent studies that have used CFA have found support for three-factor solutions [24], [39], [40]. One specific three-factor solution, reported by Dunbar et al. [21] is particularly promising, because it also has a theoretical foundation, namely the Tripartite Model of Depression and Anxiety developed by Clark and Watson [41]. The latter theoretical model assumes that anxiety and depression are separate constructs that overlap because both share a general component, namely “negative affect”. Clark and Watson have stated that this negative affect is an inherent and important aspect of both mood states [41]. In practice, Dunbar et al. have translated this theoretical model into a three-factor solution, in which the anxiety subscale of the HADS is split into a autonomous anxiety factor comprising just three items and a negative affectivity factor comprising the remaining four items originally ascribed to the anxiety subscale [21]. In addition, the negative affectivity factor is causally related to both the anxiety and depression factors [21].

Subsequently, a CTCM framework was used to identify and control for potential wording effects, by representing these wording effects as separate factors to capture response consistencies associated with wording. This approach to addressing response styles was developed by Marsh & Grayson in 1995 [34] and recently applied to analysis of the HADS by Schönberger et al. in 2010 [18]. In the CTCM framework, it is assumed that multiple psychological traits (e.g., anxiety and depression) have been measured using multiple methods (positive and negative items), and that these methods can be represented as separate factors in a CFA. For all three-factor structures, the conceptual approach thus treats method effects as a latent variable that should be incorporated into the CFA as a distinct factor in conjunction with the content factors. DiStefano and Motl have described how “the resulting relationships between items and the method factor (i.e., factor loadings) not only illustrate the strength of the relationship to the method factor, but, more importantly, allow for the method variance to be removed from the substantive construct of interest” [26]. The covariance of the method factor with the content factors (emotional distress, anxiety, depression, and negative affectivity) was constrained to be zero [18].

We computed a series of χ^2^ difference tests to compare nested models in CFA. In the current study, Razavi's one-factor model is nested within the two-factor solution of Zigmond and Snaith, because the one-factor solution can be viewed as a two-factor solution in which the correlation between factors is perfect [15], [19]. Similarly, the two-factor model is nested within the three-factor model of Dunbar et al. In the same manner, factor structures without a wording factor are nested in models with wording factors. However, one must bear in mind that we used Muthén's Weighted Least Squares with Mean and Variance Correction (WLSMV) estimator, which caused the χ^2^ values not to be χ^2^ distributed and the standard χ^2^ difference test not to be valid. For this reason, the MPlus difftest command was used to test for significant differences in model fit. Non-nested models were compared using descriptive measures such as the Comparative Fit Index (CFI), the Tucker–Lewis Index (TLI) and Root Mean Square Error of Approximation (RMSEA).

We performed some additional tests to ascertain that we were indeed dealing with item wording effects. Schönberger et al. have noted the risk that the improved fit of the factor structures, including a method factor, may be caused by the general rule that the addition of any random factor to a model can improve the model fit [18]. In accordance with Schönberger et al., we therefore test 10 additional models for each factor structure; each time, entering a random sample of eight HADS items as a factor into the analysis. In addition, previous studies have asked whether the potential method factor can be considered to be a type of response style or a substantively irrelevant artifact [26], [32]. It is has been suggested that vulnerable respondent groups (e.g. very young, less-educated individuals) may be more susceptible to the systematic wording effect, indicating the existence of a response style [25], [26], [32]. In the absence of other socioeconomic and personality characteristics, we therefore assessed the correlation of the method factor with the respondent's age and educational level.

All CFAs were computed using the statistical software package MPlus version 5, which integrates item response theory and structural equation modeling. As a result of the relative small sample size and the ordinal and non-normal nature of the scales, we employed Muthén's WLSMV as the method of estimation of the model parameters [42], [43].

## Results

### Descriptive statistics

The majority of patients interviewed were female, single and did not complete secondary school (Table 1). Mean duration of ART at the time of the interview was 37.7 days. The absolute and relative frequencies of responses to the items of the HADS are provided in Table 2. Respondents gave the highest averages scores on items 1 (“I feel tense or wound up”), 3 (“I get a sort of frightened feeling like something awful is about to happen”) and 8 (“I feel as if I am slowed down”). The total average sum score for the HADS was 10.7 (SD = 6.8), with the average sum score on the anxiety subscale and the depression subscale being 5.7 (SD = 4.0) and 4.9 (3.8), respectively. Using Zigmond and Snaith's original scoring, 15.1% of patients presented symptoms of moderate (11–14) to severe (>14) anxiety and the prevalence of moderate to severe depressive symptoms was 10.1%.

**Table 1:** Demographic and socioeconomic characteristics of the sample of 716 HIV/AIDS patients enrolled in the public-sector ART program of the Free State province, South Africa.

Sex (n (%))	Male	145 (24.0)
	Female	459 (76.0)
Age (mean (SD))		37.2 (8.9)
Marital status	Single	394 (65.2)
	Co-habiting relationship	144 (23.8)
	Not cohabiting relationship	60 (9.9)
Education (n (%))	No formal schooling	22 (3.5)
	Some primary education	132 (18.4)
	Primary education	63 (8.8)
	Some secondary education	288 (40.2)
	Grade 12	115 (16.1)
	Tertiary education	9 (1.3)
Treatment duration (mean days (SD))		37.7 (31.6)
Dwelling (n (%))	Formal	445 (73.7)
	Informal	116 (19.2)
	Traditional	38 (6.3)
	Hostel	4 (0.7)
Disability grant (n (%))	No	440 (61.5)
	Yes	189 (26.4)
HADS		
Anxiety (mean (SD))		5.69 (4.00)
Depression (mean (SD))		4.94 (3.80)
Total (mean (SD))		10.72 (6.85)
Level of anxiety (n (%))		
Absence		478 (68.7)
Mild or subclinical		113 (16.2)
Moderate		92 (13.2)
Severe		13 (1.9)
Level of depression (n (%))		
Absence		531 (74.6)
Mild or subclinical		109 (15.3)
Moderate		63 (8.8)
Severe		9 (1.3)

**Table 2:** Item score distribution of the HADS.

HADS item^1^	Percentage of answers in each answering category				Mean (SD)
	Lowest distress (0)	1	2	Highest distress (3)	
1^2^	341 (47.6)	195 (27.2)	61 (8.5)	119 (16.6)	0.94 (1.11)
2	416 (58.1)	152 (21.2)	60 (8.4)	88 (12.3)	0.75 (1.04
3^2^	353 (49.3)	131 (18.3)	163 (22.8)	69 (9.6)	0.93 (1.05)
4	509 (71.3)	127 (17.8)	54 (7.6)	24 (3.4)	0.43 (0.77)
5^2^	379 (54.1)	192 (27.4)	83 (11.9)	49 (7.0)	0.72 (0.93)
6	423 (59.1)	144 (20.1)	110 (15.4)	39 (5.4)	0.67 (0.93)
7	451 (63.0)	122 (17.0)	119 (16.6)	24 (3.4)	0.60 (0.88)
8^2^	257 (35.9)	347 (48.5)	71 (9.9)	40 (5.6)	0.85 (0.81)
9^2^	337 (47.1)	260 (36.3)	83 (11.6)	35 (4.9)	0.74 (0.85)
10^2^	463 (64.8)	67 (9.4)	68 (9.5)	117 (16.3)	0.77 (1.16)
11^2^	366 (51.3)	181 (25.4)	74 (10.4)	92 (12.9)	0.85 (1.05)
12	364 (50.9)	137 (19.2)	74 (10.3)	140 (19.6)	0.99 (1.18)
13^2^	311(43.6)	283 (39.6)	74 (10.4)	46 (6.4)	0.80 (0.87)
14	533 (74.6)	79 (11.1)	45 (6.3)	57 (8.0)	0.48 (0.93)

1 Zigmond & Snaith's originally stipulated that items with unequal numbers are part of the anxiety subscale and items with equal numbers are part of the depression subscale.

2 Negatively worded items.

### Examination of individual subscales

Before assessing and comparing the model fit of the different factor solutions, we assessed the model fit of the different individual subscales (seven-item anxiety subscale, seven-item depression subscale, three-item anxiety subscale, and four-item negative affectivity factor). The one-factor solution did not comprise any subscales and is therefore discussed in the next paragraph.

A confirmatory factor analysis was performed to assess the model fit of the seven-item anxiety subscale of the two-factor solution of Zigmond and Snaith. A χ^2^ goodness of fit test revealed that the omnibus test of the model did not fit the given data (χ^2^ = 25.856, df = 14, p<0.05). However, because even small amounts of residual covariance are often significant with relatively large samples, the χ^2^ test statistics are almost certainly significant, even for good-fitting models [44]; and the test is thus very difficult to pass in analyses based on large samples and in models containing many observed variables [44]. Therefore, and in accordance with methodological recommendations, additional descriptive fit measures should be and are reported throughout the remainder of this analysis [45]. These measures (RMSEA, CFA, TLI) revealed a good model fit (Table 3). All factor loadings were significant and ranged from 0.376 (item 1) to 0.795 (item 13). Using Raykov's latent variable modeling procedure for evaluating the reliability of a scale, we calculated the composite reliability, which was acceptable at 0.702 [46]. A similar CFA of the seven-item depression factor revealed a good model fit (CFI = 0.980, RMSEA = 0.045). Again all factor loadings were significant (p<0.001). Item 10, stating “I have lost interest in my appearance”, displayed the lowest loading (0.264) and item 4 the highest loading (0.699). The composite reliability of the subscale was 0.628. We subsequently performed a CFA to assess independently the goodness-of-fit of the autonomous anxiety subscale of the three-factor solution of Dunbar et al. The descriptive measures indicate an excellent model fit (CFI = 0.999, RMSEA = 0.000). All factor loadings were significant and sufficiently high. Composite reliability of the subscale was 0.707. Finally, a CFA was performed on the negative affectivity subscale of the three-factor structure. The factor had a satisfying fit to the data (CFI = 0.981, RMSEA = 0.043). Again, all factor loadings were highly significant (p<0.001), with item 1 displaying the lowest loading (0.369) and item 5 the highest (0.619). Composite reliability of this subscale was rather low at 0.466.

**Table 3:** Standardized factor loadings of the HADS items and goodness of fit statistics for all six alternative factor structures.

	Model 1: One-factor model	Model 2: Two-factor model		Model 3: Three-factor model			Model 4: One-factor model with negative wording		Model 5: Two-factor model with negative wording			Model 6: Three-factor model with negative wording			
HADS items	Emotional distress	Anxiety	Depression	Autonomous anxiety	Depression	Negative affectivity	Emotional distress	Negative wording	Anxiety	Depression	Negative wording	Autonomous anxiety	Depression	Negative affectivity	Negative wording
1	0.390	0.402				0.381	0.265	0.329	0.267		0.329			0.471	0.186
2	0.581		0.623		0.623		0.658			0.659			0.595		
3	0.634	0.657		0.697			0.416	0.539	0.421		0.536	0.558			0.409
4	0.567		0.611		0.614		0.657			0.658			0.666		
5	0.594	0.614				0.580	0.504	0.325	0.510		0.319			0.577	0.300
6	0.658		0.702		0.700		0.734			0.735			0.752		
7	0.526	0.539				0.517	0.598		0.602					0.648	
8	0.590		0.628		0.626		0.468	0.380		0.486	0.384		0.485		0.449
9	0.728	0.754		0.797			0.481	0.594	0.486		0.589	0.639			0.455
10	0.316		0.330		0.333		0.246	0.213		0.246	0.216		0.272		0.208
11	0.494	0.511				0.484	0.420	0.269	0.424		0.264			0.473	0.252
12	0.519		0.561		0.560		0.600			0.601			0.609		
13	0.714	0.740		0.784			0.409	0.709	0.414		0.706	0.557			0.594
14	0.442		0.472		0.475		0.510			0.510			0.512		
Composite reliability															
	0.702	0.702	0.628	0.707	0.628	0.466									
Goodness of fit															
RMSEA	0.080		0.073			0.062		0.039			0.040				0.056
CFI	0.904		0.922			0.945		0.979			0.979				0.960
TLI	0.887		0.907			0.932		0.973			0.971				0.946

### Fit of the one-, two- and three-factor models

**Figure 1:**
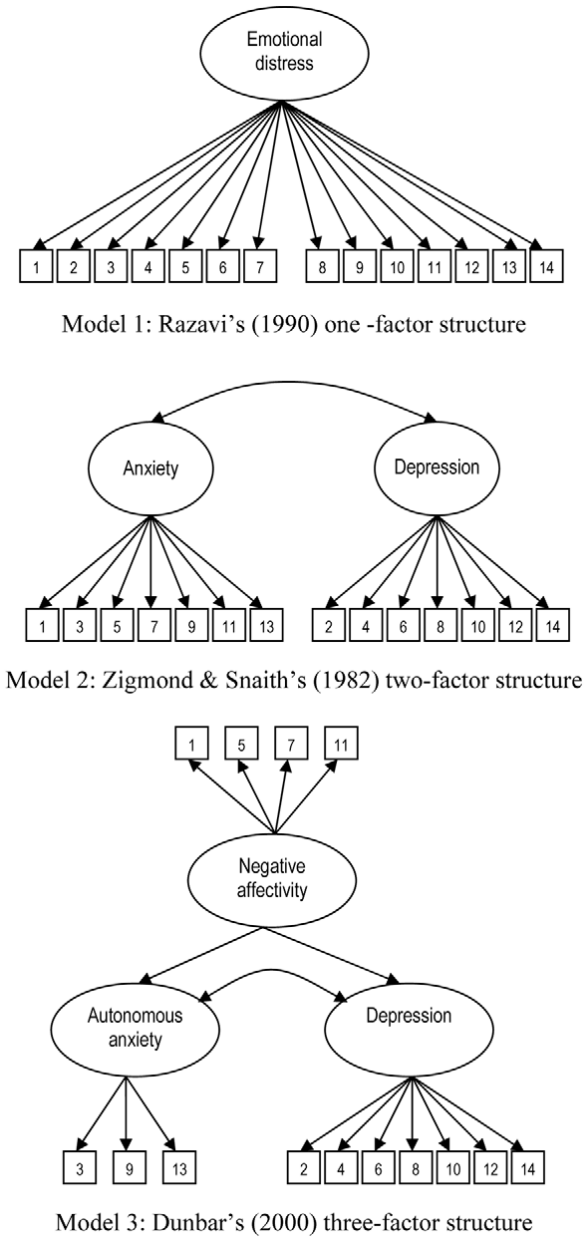
Overview of the three alternative factor structures of the HADS, supported by the literature.

CFA was used to assess the fit of the three alternative factor structures of the HADS (Table 3 & Figure 1). Model 1 – the one-factor solution postulated by Razavi et al.– provided a borderline acceptable fit to the data. A χ^2^ goodness of fit test revealed that the model did not fit the given data (χ^2^ = 430.391, df = 77, p<0.001), but – as mentioned above – it is recommended to rely on alternative fit indices in the evaluation of model fit [45]. The CFI of 0.904 indicated a reasonable model fit and the TLI of 0.887 and RMSEA of 0.080 indicated a borderline acceptable fit of the model to the data. If we look at the factor loadings, Table 3 demonstrates that all loadings on the single emotional distress factor were highly significant (p<0.001). Standardized factor loadings ranged from 0.728 (item 9) to 0.316 (item 10). The composite reliability of the one-factor solution was good (0.702).

A CFA on the original two-factor structure (Model 2) revealed an acceptable fit of the model to the data, as indicated by the different goodness-of fit measures (RMSEA = 0.073; CFI = 0.922; TLI = 0.907). The χ^2^ test again did not support the model. The estimated correlation between the anxiety and depression subscales was 0.811 (p<0.001). All factor loadings were highly significant (p<0.001). When looking at the anxiety subscale, the standardized factor loadings ranged from 0.402 (item 1) to 0.754 (item 7). The correlation between the seven items measuring depression and the factor ranged from 0.330 (item 10) to 0.702 (item 6). As indicated above, the composite reliability of both subscales was acceptable.

The results of the CFA indicate that the three-factor model (model 3) – the Hierarchical Tripartite Model of Dunbar et al. – fits the data well. Both descriptive indices indicated a good model fit (RMSEA = 0.065: CFI = 0.938; TLI = 0.932). Again, all factor loadings were significant (p<0.001). The three-item autonomous anxiety subscale displayed factor loadings ranging from 0.697 (item 3) and 0.797 (item 9). The composite reliability of the three-item subscale was 0.707. Six items loaded sufficiently on the depression factor, and only item 10 was only weakly linked to the depression factor (0.333). As indicated above, the estimation of the composite reliability of the depression subscale indicated satisfactory reliability. When investigating the Negative Affectivity subscale, the results of the CFA indicated that all standardized factor loadings were positive and highly significant (p<0.001), ranging from 0.381 (item 1) to 0.580 (item 5). The subscale had rather low composite reliability. The correlation between the depression factor and the autonomous anxiety factor was 0.682. The correlation between the negative affectivity factor and the depression and autonomous anxiety factors was high, amounting to 0.841 and 0.798 respectively.

We compared the fit of the three different factor structures using χ^2^ difference testing. The one-factor model had a significantly poorer fit than both the two- and the three-factor models, and the two-factor model had a poorer fit than the three-factor model. However, as indicated above, the differences in model fit between the three models were only marginal, as shown by the similar RMSEA, CFI and TLI values.

### Adding a wording factor

**Figure 2:**
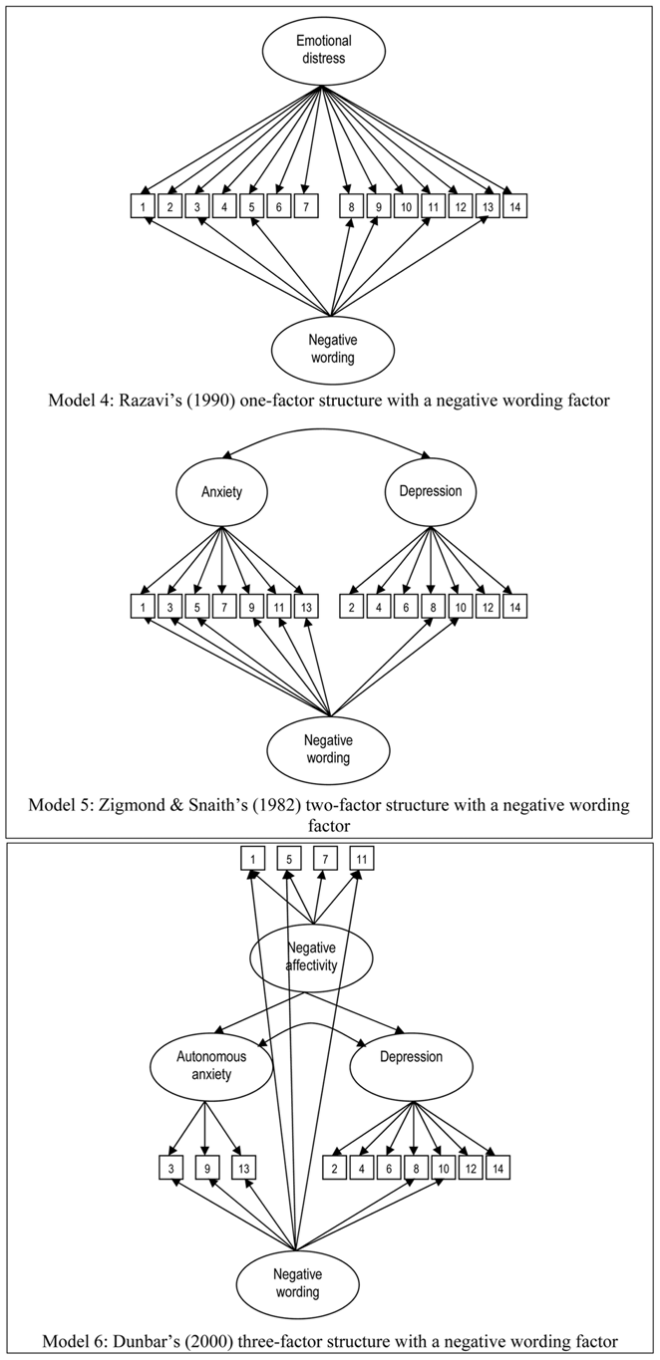
Overview of the three alternative factor structures of the HADS, supported by the literature including a negative wording factor.

We subsequently estimated three additional models to examine the presence of wording effects in the measurement of the HADS (Figure 2). In accordance with previous studies, we examined whether adding a negative wording factor significantly improved the fit of the model (Models 4–6) (Table 3) [18], [36], [47]. Eid et al. (2000) named this the *correlated trait-correlated method minus 1* (CTCM-1) because one method is the standard of comparison for which no method factor will be specified [47].

Model 4 added a negative wording factor to the one factor solution of Razavi et al. The model displayed a good fit to the data, as indicated by the descriptive indices (RMSEA = 0.039; CFI = 0.979; TLI = 0.973). The model with method factor (model 4) fitted the data significantly better than the one-factor solution without a negative wording factor (Model 1) (p<0.001). All factor loadings were positive and highly significant (p<0.001). However, items 1 and 10 did not sufficiently load onto the combined anxiety/depression factor (standardized factor loadings of 0.265 and 0.246, respectively). All negative items loaded significantly on the negative wording factor (p<0.001).

Addition of a negative wording factor to the two-factor structure resulted in a good model fit (Model 5), as indicated by the descriptive measures (RMSEA = 0.040; CFI = 0.979; TLI = 0.971). When we compared the fit of the two-factor model without a method factor to that of Model 4 – i.e. the two-factor solution including a negative wording factor – the RMSEA, CFI, TLI and the χ^2^ difference test all indicated that the latter fitted the data better than the two-factor model without a method factor. Looking at the seven-item depression subscale, all factor loadings were again highly significant (p<0.001) and only the standardized loading of item 1 was low (0.267). Similarly, all seven items loaded significantly on the anxiety factor, and only item 10 again displayed a low standardized factor loading (0.246). The correlation between the anxiety and depression factors was 0.984 (p<0001). Again, all items loaded highly significantly on the method factor (p<0.001).

The Tripartite Model with negative item wording factor (Model 6) showed a good fit to the data (RMSEA = 0.056; CFI = 0.960, TLI = 0.946). Again, all indicators demonstrated that the factor solution with a method factor was significantly better than a similar factor structure without the negative wording factor (p<0.001). For all three subscales – negative affectivity, autonomous anxiety and depression – all factor loadings were highly significant, and all but item 10 (standardized factor loading = 0.272) loaded sufficiently on their respective factor. The correlations between the negative affectivity factor and the autonomous anxiety and depression factors were 0.765 and 0.792, respectively. The correlation between the three-item autonomous anxiety factor and the depression factor was 0.740.

We compared the fit of three factor structures with a method factor (Models 4–6). Both the descriptive measures (RMSEA, CFI and TLI) and the χ^2^ difference tests demonstrated that the two- and three-factor solutions with a method factor certainly did not fit the data better than Model 4; the one-factor solution with a negative wording factor. The descriptive indices were similar for all three models and the χ^2^ difference testing revealed no significant differences. The results indicated that the one-factor solution with a negative wording factor fitted the data best. It must be noted that the addition of a method factor significantly (p<0,001) improved the fit of the one-, two-, and three-factor solutions.

Following the procedure of Marsh and Grayson [36] and recently applied by Schönberger et al. [18], we subsequently examined whether adding a positive wording factor – creating a full CTCM model – resulted in an even better fit of the different factor solutions to the data. The addition of a second positive wording factor to the three-factor structures repeatedly resulted in a non-admissible solution (latent variable covariance matrix was not positive definite).

### Additional analyses

Schönberger et al. have noted the risk that the improved fit of the factor structures including a method factor may be caused by the general rule that addition of any random factor to a model can improve the model fit [18]. To assess whether we were really dealing with an item wording factor or we just found a better fit simply because of the addition of a random factor, we executed 30 additional CFAs (10 for each factor structure), in which we replaced the method factor by a random sample of eight HADS items. The majority of these models (*n = *22) could not be identified. The remaining eight models all had a worse fit to the data – as indicated by the RMSEA, CFI and TLI – compared to the corresponding model with the item wording factor. In none of these models were the loadings on the method factor consistently significant and positive/negative.

We subsequently calculated the correlation between the negative wording factor and the respondents' age and educational level. In none of the three-factor structures did we find a significant correlation between the item wording factor and the age of the HIV/AIDS patients. The correlation between the negative wording factor and the education level of the respondents was consistently negative and borderline significant. The correlation ranged from −0.119 (p = 0.051) for the one-factor solution, to −0.120 (p = 0.051) and −0.150 (p = 0.052) for the two- and three-factor solutions, respectively.

## Discussion

The aims of this study were to: (1) examine the factor structure of the HADS in a population of HIV/AIDS patients enrolled in the South African public sector ART program; and (2) identify and control for the disturbing influence of systematic wording effects in vulnerable respondent groups. Although estimates of the prevalence of anxiety and depression in similar populations differ widely, the levels of anxiety and depression reported in our population of South African ART patients fell in the middle range: the prevalence of anxiety and depression was considerably higher than that reported in an Ethiopian population [12], similar to that in other studies performed in Hong Kong [48] and the United Kingdom [49] and considerably lower than that reported in a Brazilian study [50]. One must note however, that all studies that reported significantly higher levels of anxiety and depression were conducted on patients who did not have access to ART. The only two studies that explicitly selected ART patients have reported lower or similar levels of anxiety and depression [12], [49].

When assessing the factor structure without a negative wording factor, it is clear that all three-factor structures displayed an acceptable fit to the data. However, the fit of the one-factor solution was significantly poorer than that of the two- and three-factor solutions, and in its turn, the original two-factor solution displayed a significantly poorer fit than the three-factor model of Dunbar et al [21]. The superior fit of the Tripartite Model seems to support the underlying theory of anxiety and depression developed by Clark and Watson in 1991 [41]. In this regard, our study findings contradict those of Reda in 2011 [12] – the only previous scientific study assessing the factor structure of the HADS in a population of ART patients in a developing country with high HIV prevalence. This author has reported that the HADS has a single underlying dimension, as indicated by Razavi's model [19]. We must note however, that the differences in model fit between the three solutions, observed in our study, were only minor. In addition, the high correlations between the different latent factors in both the two- and the three-factor solutions raise the question whether the HADS is really a multidimensional scale.

The introduction of a method factor identifying and controlling for the negative wording effect improved the model fit significantly. A combined CFA and CTCM-1 framework demonstrated that the wording effects associated with negatively worded items in the HADS could be estimated as a distinct latent variable. The addition of this method factor significantly improved the fit of the one-, two-, and three-factor solutions. This supports the study findings of Schönberger et al. [18], who have indicated that all three-factor structures with negative item wording displayed a superior fit compared to even the best fitting factor structure without a method factor. The consistent improvements in model fit raise the question of how the poor model fit of different factor structures in other studies – without a negative wording factor – could have been improved by controlling for item wording effects.

However, Horan et al. [32] and DiStefano et al. [26] have rightfully asked whether the observed method factor can be considered to be a type of response style or a substantively irrelevant artifact. The current study tentatively explored the nature of the method effect by relating it to the age and educational level of respondents. We found that the association between the level of schooling and the method factor of negative wording was borderline significant, indicating that less-educated respondents were more susceptible to this response style. This is in line with previous studies on item wording factors by Chen et al. [51] and Schmitt and Allik [31], who have indicated that higher levels of schooling cause respondents to treat negatively and positively worded items more equally. In addition, it has been demonstrated that wording of positive and negative items is much more influential in developing and more unequal societies [31]. Taken together, these findings demonstrate that item wording effects should be taken into account when applying the HADS to vulnerable populations such as ART patients in high-HIV-prevalence, resource-limited settings. However, further research is needed to establish why different populations respond differently to negatively and positively worded items, and which socio-demographic and personality characteristics are associated with this response style [25], [26], [31].

We used CFA to evaluate the competing structures underlying the HADS while controlling for item wording effects, and demonstrated that Razavi's one-factor solution best fitted the data [19]. The addition of content factors to create Zigmond and Snaith's two-factor solution and the three-factor solution of Dunbar et al. only worsened the model fit [15], [21]. These outcomes are remarkable, because an investigation into the factor structure of the HADS without an item method factor would have rejected Reda's preference for the one-factor solution, while the superior fit of Razavi's model with a negative item wording factor strongly supports the recent study findings [12]. One must note however, that all three-factor structures including the method factor displayed a good fit to the data. In particular, the difference between the one- and two-factor models with a method factor was very small, indicating that the original two-factor solution can also be applied when screening public-sector ART patients for symptoms of anxiety and depression. The choice for a particular factor solution should therefore not only depend on statistical arguments, but also on the theoretical underpinnings of the research, the purpose of the testing, and the population under investigation [18].

One item deserves additional attention. In all analyses, Item 10 (“I have lost interest in my appearance”) displayed a low contribution to both the general emotional distress and specific depression factors. This agrees with previous studies indicating that this item only weakly correlates with the theoretically derived constructs [52]–[56]. Matsudaira et al. have indicated that the item may be influenced by a latent factor other than depression, such as interpersonal attraction or social desirability [56]. Further investigation is thus needed to identify the confounding factors of Item 10 of the HADS.

The strengths of this study included the application of the combined CFA and CTCM approach to an increasingly relevant topic (the growing dual epidemic of HIV and mental health problems) and the availability of information on an understudied population (716 HIV/AIDS patients from a developing country). To the best of our knowledge, this is only the second study to assess the factor structure of the HADS in a sample of HIV/AIDS patients on ART in sub-Saharan Africa [12], and the first to include a method factor. However, there were some limitations to our study. First, the study findings provided a clear indication of the presence of a response style reflected in a systematic pattern of responses to negatively worded questionnaire items. However, more research is needed to demonstrate definitively the existence of a negative wording method effect because this is only one possible explanation for the occurrence of this systematic pattern of variance. There are other confounding factors – the content area under study, personality factors of the respondents, characteristics of the scaling method – that might exert an influence on item responses [25]. The tendency to respond differently to positively and negatively worded items has been shown to be dependent on an individual's level of approach motivation and psychological adjustment, or being more susceptible to social desirability response effects [26], [33], [57], [58]. However, this information was not available in our dataset. Although we showed that the method factor was correlated with the educational level of the respondents, more work needs to be done to identify additional personality, demographic and methodological factors to explain method effects associated with negative phrasing. Second, the current study did not include a diagnostic assessment, rendering the HADS the only available psychiatric measure. The main aim of the FEATS project was much broader than psychiatric outcomes, limiting the relative weight of psychiatric measures in the overall questionnaire. The current study was interested in screening for symptoms of anxiety and depression, which justifies the use of the HADS, an instrument which has been validated in South Africa in non-HIV-positive and non-psychiatric populations with adequate psychometric properties [59], [60]. Finally, the fit of the different factor structures and the impact of negative wording on the item responses may not be applicable to alternative settings. We can only ascribe the findings to patients enrolled in a public sector ART program and, more specifically, to patients enrolled in South Africa's Free State province ART program. Large-scale studies investigating the applicability of the HADS to this particularly vulnerable population are urgently needed.

The study findings have both theoretical and practical implications. From a theoretical point of view, the CFA results demonstrate that all three-factor structures with a theoretical foundation displayed an acceptable fit to the data. The identification and correction for negative wording effect, however, resulted in a superior fit for Razavi's one-factor solution with the HADS as a single measure of emotional distress. From a practical perspective, the CFA results support the use of the HADS as a valid and reliable means to screen for mental health problems in HIV/AIDS patients enrolled in a public-sector ART program in a resource-limited context. The availability of such a trustworthy instrument to assess rapidly the mental health of each patient is vital given the immense burden that HIV/AIDS and the associated antiretroviral care are putting on the health system. In addition, the results demonstrate the importance of evaluating and correcting for wording effects when examining the factor structure of this screening instrument. This is especially important when assessing the mental health of vulnerable patient groups in high-HIV-prevalence developing countries. Researchers have suggested practical alternatives to positively and negatively phrased items to guard against acquiescence: instead of mixing the item phrasing, the response options could be reversed or, sum scores could only include the positively worded items to avoid lowering sum or mean scores [26], [61], [62]. In light of the inter-relationships between HIV/AIDS and mental health problems and the scarcity of adequate screening tools, additional studies need to be conducted to explore further the factor structure of the HADS in high-HIV-prevalence, resource-limited settings, while assessing and controlling for potential response styles in these vulnerable populations.
